# Supplementary material for: A statistical resolution measure of fluorescence microscopy with finite photons
Source: Nat Commun. 2024 May 4;15:3760. doi: 10.1038/s41467-024-48155-x (PMC11069581; doi:10.1038/s41467-024-48155-x)
Supplement: Supplementary file 1 — Supplementary Information [file 41467_2024_48155_MOESM1_ESM.pdf]

Supplementary Materials for

A statistical resolution measure of fluorescence microscopy with finite photons

Yilun Li[1], Fang Huang[1,2,3]*

Correspondence to: fanghuang@purdue.edu

This PDF file includes:

Supplementary Text: Note1-Note4

Supplementary Figs. 1-13

Supplementary Text

Note 1: Resolution criterion threshold on information density

The information-based resolution (IbR) is defined as the reciprocal of frequency where information density I_d drops below 10 rad^{-2}μm^{-2} threshold. For an object of $k = \frac{NA}{\lambda}$, applying threshold $I_d = 10\ rad^{-2}\mu m^{-2}$ to a unit area (one cycle of the sinusoidal wave), the Fisher information corresponds to phase estimation uncertainty $\sigma_\phi = \frac{\pi}{2.2}$ (**Fig. S2**). Since $\frac{\pi}{2}$ phase difference can switch sinusoidal pattern to cosine pattern, phase estimation uncertainty of $\sigma_\phi = \frac{\pi}{2.2}$ can be a critical point used as a threshold distinguishing resolved and unresolved.

Note 2: Data post-processing influence on Fisher information

Supposing we have 2 random variables which are J and K denoting raw data and processed data. K is determined by J by function $K = H(J)$ where H is a deterministic function. Consider the chain rule of Fisher information (*44*)

$$I_{J,K}(\theta) = I_J(\theta) + I_{K|J}(\theta).$$

The conditional Fisher information is

$$I_{K|J}(\theta) = E_j[I_{K|J=j}(\theta)].$$

By Fisher information definition we have

$$I_{K|J=j}(\theta) = E_k[\left(\frac{\partial \log(p(K|J=j;\theta))}{\partial\theta}\right)^2],$$

where $p(K|J = j; \theta)$ is the conditional probability density function. Since $K = H(J)$ is determined as

$$p(K|J = j; \theta) = \begin{cases} 1 & K = H(j) \\ 0 & otherwise \end{cases},$$

above equation indicates the conditional probability density function is determined on condition but irrelevant to any parameter θ. Thus, the partial derivative is zero resulting in

$$I_{K|J=j}(\theta) = 0, \forall j,$$

$$I_{K|J}(\theta) = E_j[I_{K|J=j}(\theta)] = 0.$$

Combining first equation we have

$$I_J(\theta) = I_{J,K}(\theta) = I_K(\theta) + I_{J|K}(\theta) \geq I_K(\theta).$$

Last step of inequality comes from fact that any Fisher information is greater or equal to 0. If the processing is deterministic, then Fisher information in the processed data K would always be smaller or equal to raw data J. In another word, deterministic image post-processing cannot increase Fisher information of any parameter. Note that this statement is under condition that image processing is deterministic. If other prior information could be accessed, this relationship would not stand.

Note 3: Pixel binning effect on image

The pixel binning effect is well known for functioning as low pass filter (*33*). We derive the pixel binning effect in mathematical formula as follows.

Supposing we have a 2D image with coordinate (x, y), the image is recorded with discrete pixel index $[n, m]$. The function of each individual pixel is to integrate all photons falls within the pixel

$$Img[n,m] = \int_{x_n-\frac{d}{2}}^{x_n+\frac{d}{2}} \int_{y_m-\frac{d}{2}}^{y_m+\frac{d}{2}} Img(x,y)dxdy.$$

Here (x_n, y_m) is the coordinate which is at the center of pixel $[n, m]$ and d is the pixel size. $Img(x, y)$ is photon distribution function. $Img[n, m]$ is the image value at each pixel. We create a function defined as

$$f(x,y) = \frac{1}{d^2} \int_{x-\frac{d}{2}}^{x+\frac{d}{2}} \int_{y-\frac{d}{2}}^{y+\frac{d}{2}} Img(x',y')dx'dy'.$$

Then

$$Img[n,m] = f(x_n, y_m)d^2.$$

Applying Fourier transform on function $f(x, y)$ we have

$$\hat{f}(\xi,\eta) = \frac{1}{d^2} \iint_{-\infty}^{+\infty} f(x,y)e^{-2\pi i x\xi} e^{-2\pi i y\eta} dxdy$$

$$= \frac{1}{d^2} \iint_{-\infty}^{+\infty} [\int_{x-\frac{d}{2}}^{x+\frac{d}{2}} \int_{y-\frac{d}{2}}^{y+\frac{d}{2}} Img(x',y')dx'dy'] e^{-2\pi i(y\eta+x\xi)} dxdy.$$

Let $x' = x + \delta x$, $y' = y + \delta y$, we have

$$= \frac{1}{d^2} \iint_{-\infty}^{+\infty} [\int_{-\frac{d}{2}}^{\frac{d}{2}} \int_{-\frac{d}{2}}^{\frac{d}{2}} Img(x+\delta x, y+\delta y) d\delta x d\delta y] e^{-2\pi i(y\eta+x\xi)} dxdy.$$

Exchange the integration sequence we have

$$= \frac{1}{d^2} \int_{-\frac{d}{2}}^{\frac{d}{2}} \int_{-\frac{d}{2}}^{\frac{d}{2}} [\iint_{-\infty}^{+\infty} Img(x+\delta x, y+\delta y) e^{-2\pi i(y\eta+x\xi)} dxdy] d\delta x d\delta y$$

$$= \frac{1}{d^2} \int_{-\frac{d}{2}}^{\frac{d}{2}} \int_{-\frac{d}{2}}^{\frac{d}{2}} \widehat{Img}(\xi,\eta) e^{2\pi i(\delta y\eta+\delta x\xi)} d\delta x d\delta y$$

$$= \widehat{Img}(\xi,\eta) \frac{\sin(\pi\xi d)}{\pi\xi d} \frac{\sin(\pi\eta d)}{\pi\eta d}.$$

From expression, function $f(x, y)$ is function $Img(x, y)$ frequency filtered by two sinc functions. The image pixels' value $Img[n, m]$ is the sampling on function $f(x_n, y_m)$. Pixel size d not only affects the sampling process but also affects the frequency filter applied on the image.

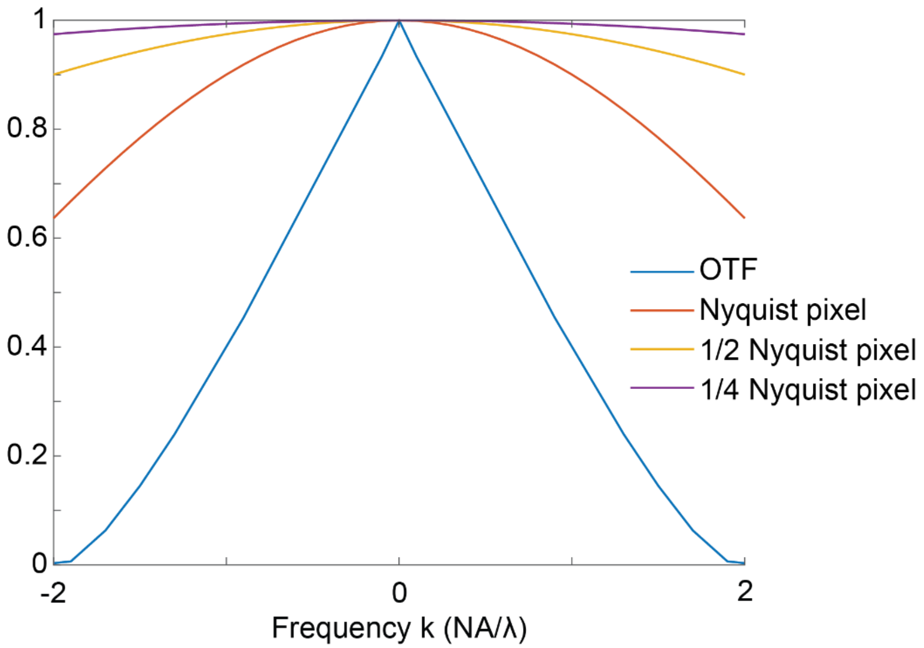

OTF and low pass filter by pixelization in 1d. When the pixel size is at the Nyquist sampling requirement: $d = \frac{\lambda}{4NA}$, the transmission rate at $k = \frac{2NA}{\lambda}$ is $\frac{\sin(\pi * \frac{2NA}{\lambda} * \frac{\lambda}{4NA})}{\pi * \frac{2NA}{\lambda} * \frac{\lambda}{4NA}} = \frac{2}{\pi} \approx 0.637$ (red curve).

Note 4: Effective OTF of confocal

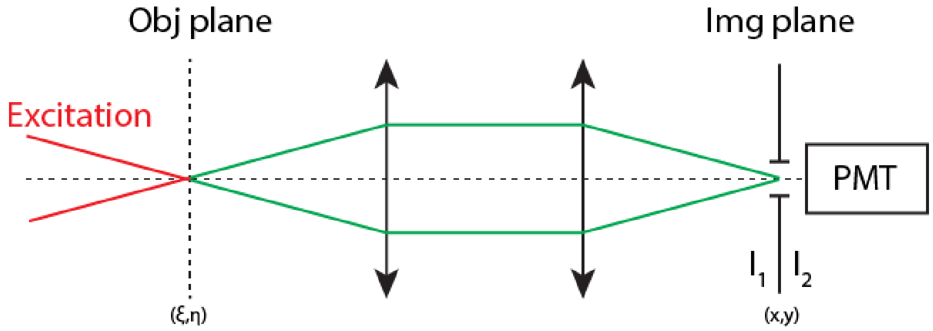

Diagram of a confocal microscope.

The OTF derivation of confocal can be found in works (*23*,*40*). For completeness and reference, we provide our version of derivation below.

The fluorescence distribution on the object plane is

$$I_{obj}(\xi,\eta) = obj(\xi,\eta) \cdot PSF_{ex}(\xi - \xi_0, \eta - \eta_0) \cdot \kappa,$$

where $obj(\xi,\eta)$ is the fluorophore density distribution, $PSF_{ex}(\xi,\eta)$ is the excitation PSF, (ξ_0,η_0) denotes the scanning position and κ is the excitation to emission coefficient we assumed to be 1 for simplicity. We also assumed the magnification is 1 for simplicity.

Forming the ideal image before pinhole we have,

$$I_1(x,y) = \iint_{-\infty}^{+\infty} PSF(x-\xi, y-\eta) I_{obj}(\xi,\eta) d\xi d\eta.$$

The ideal image after pinhole would be,

$$I_2(x,y) = I_1(x,y) \cdot I_{ph}(x-x_0, y-y_0),$$

where $I_{ph}(x,y)$ is the pinhole function either zero or one, (x_0, y_0) is the conjugate position of scanning position on the image plane. Since magnification is 1, the conjugate relationship would be $(x_0, y_0) = (-\xi_0, -\eta_0)$.

In confocal imaging, a bucket photon detector would collect all photons for each scanning position and form a value. Therefore, the ideal image value of confocal at (x_0, y_0) would be,

$$I_{img}(x_0,y_0) = \iint_{-\infty}^{+\infty} I_2(x,y) dxdy$$

$$= \iint_{-\infty}^{+\infty} I_1(x,y) \cdot I_{ph}(x-x_0, y-y_0) dxdy$$

$$= \iint_{-\infty}^{+\infty} [\iint_{-\infty}^{+\infty} PSF(x-\xi, y-\eta) I_{obj}(\xi,\eta) d\xi d\eta] \cdot I_{ph}(x-x_0, y-y_0) dxdy.$$

Exchange the integration order we have,

$$= \iint_{-\infty}^{+\infty} [\iint_{-\infty}^{+\infty} PSF(x-\xi, y-\eta) I_{ph}(x-x_0, y-y_0) dxdy] \cdot I_{obj}(\xi,\eta)\, d\xi d\eta.$$

If we assume the pinhole function is symmetric, we have,

$$= \iint_{-\infty}^{+\infty} [\iint_{-\infty}^{+\infty} PSF(x-\xi, y-\eta) I_{ph}(x_0-x, y_0-y) dxdy] \cdot I_{obj}(\xi,\eta)\, d\xi d\eta.$$

Let $x' = x - \xi$ and $y' = y - \eta$, the equation becomes,

$$= \iint_{-\infty}^{+\infty} [\iint_{-\infty}^{+\infty} PSF(x', y') I_{ph}(x_0+\xi-x', y_0+\eta-y') dx'dy'] \cdot I_{obj}(\xi,\eta)\, d\xi d\eta.$$

The integration in the bracket above is considered as a convolution between PSF and I_{ph} taking the value at $(x_0+\xi, y_0+\eta)$, then the equation becomes,

$$= \iint_{-\infty}^{+\infty} (PSF \otimes I_{ph})(x_0+\xi, y_0+\eta) \cdot I_{obj}(\xi,\eta)\, d\xi d\eta.$$

Consider the conjugate relationship $(x_0, y_0) = (-\xi_0, -\eta_0)$ and we have,

$$= \iint_{-\infty}^{+\infty} (PSF \otimes I_{ph})(\xi-\xi_0, \eta-\eta_0) \cdot I_{obj}(\xi,\eta)\, d\xi d\eta.$$

Expand the fluorescence distribution expression we have,

$$= \iint_{-\infty}^{+\infty} (PSF \otimes I_{ph})(\xi-\xi_0, \eta-\eta_0) \cdot PSF_{ex}(\xi-\xi_0, \eta-\eta_0) \cdot obj(\xi,\eta)\, d\xi d\eta$$

$$= \iint_{-\infty}^{+\infty} (PSF \otimes I_{ph} \cdot PSF_{ex})(\xi-\xi_0, \eta-\eta_0) \cdot obj(\xi,\eta)\, d\xi d\eta.$$

If we consider PSF and PSF_{ex} are also symmetric, the equation becomes,

$$= \iint_{-\infty}^{+\infty} (PSF \otimes I_{ph} \cdot PSF_{ex})(\xi_0-\xi, \eta_0-\eta) \cdot obj(\xi,\eta)\, d\xi d\eta.$$

Writing in this expression allows us to identify the ideal image formation in confocal as object convolution with an effective PSF where,

$$PSF_{eff} = PSF \otimes I_{ph} \cdot PSF_{ex}.$$

The corresponding effective OTF would be Fourier transform on the PSF expression,

$$OTF_{eff} = OTF \cdot FT\{I_{ph}\} \otimes FT\{PSF_{ex}\}.$$

Figures of effective OTF of confocal with different pinhole diameters are plotted in Fig. S11.

Note 5: Conceptual differences between IbR and FRC

Information based resolution (IbR) is different from any Fourier Ring Correlation (FRC) defined resolution method, where FRC is directly computed from an observed image (*13, 14*). In contrast, our Fisher information density is a theoretical measure based on the imaging conditions, independent of specific observed image. The noisy images we added to the figures in manuscript are simulated noisy images for visualization only. They are not used for our Fisher information calculation. Fisher information is purely based on **deterministic** theoretical calculation and thus the information density or IbR can be considered as a function of system and sample parameters.

$$I(\phi) = \text{information}(n, NA, \lambda, photon, k, \text{modality}, d, \text{bg})$$

Where n is refractive index, NA is the numerical aperture, λ is the emission wavelength, $photon$ is the emitted photons from target object, k is the frequency of the object, modality represents a particular microscopy design, d is the volume thickness, bg is the background fluorescence density. In contrast, FRC is a post-acquisition resolution evaluation on a specific noisy image.

$$Correlation = FRC(\text{acquired\_image})$$

The meaning of our theoretical Fisher information measure for resolving power is that it provides a potential way to compare different imaging modalities theoretically in a statistical manner. In Abbe's resolution consideration, resolution is only affected by numerical aperture NA and wavelength λ. Our information density allows us to rewrite resolution as a function of the emitted photons.

Note 6: Photon collection efficiency

The emitter in the sample plane is emitting photons in a 4π solid angle. Photon collection efficiency is calculated by the ratio between solid angle limited by the aperture of the microscope and 4π. By utilizing a spherical surface model we can calculate

$$efficiency = \frac{S}{4\pi r^2} = \frac{\int_0^{\theta_0}\int_0^{2\pi} r^2 \sin(\theta) d\phi d\theta}{4\pi r^2} = \frac{1-\cos(\theta_0)}{2},$$

Where angle θ_0 is calculated from $NA = n\sin(\theta_0)$.

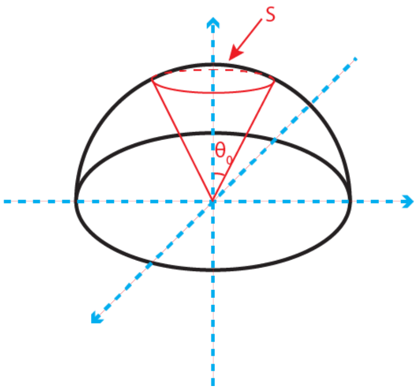

Solid angle photon collection efficiency diagram.

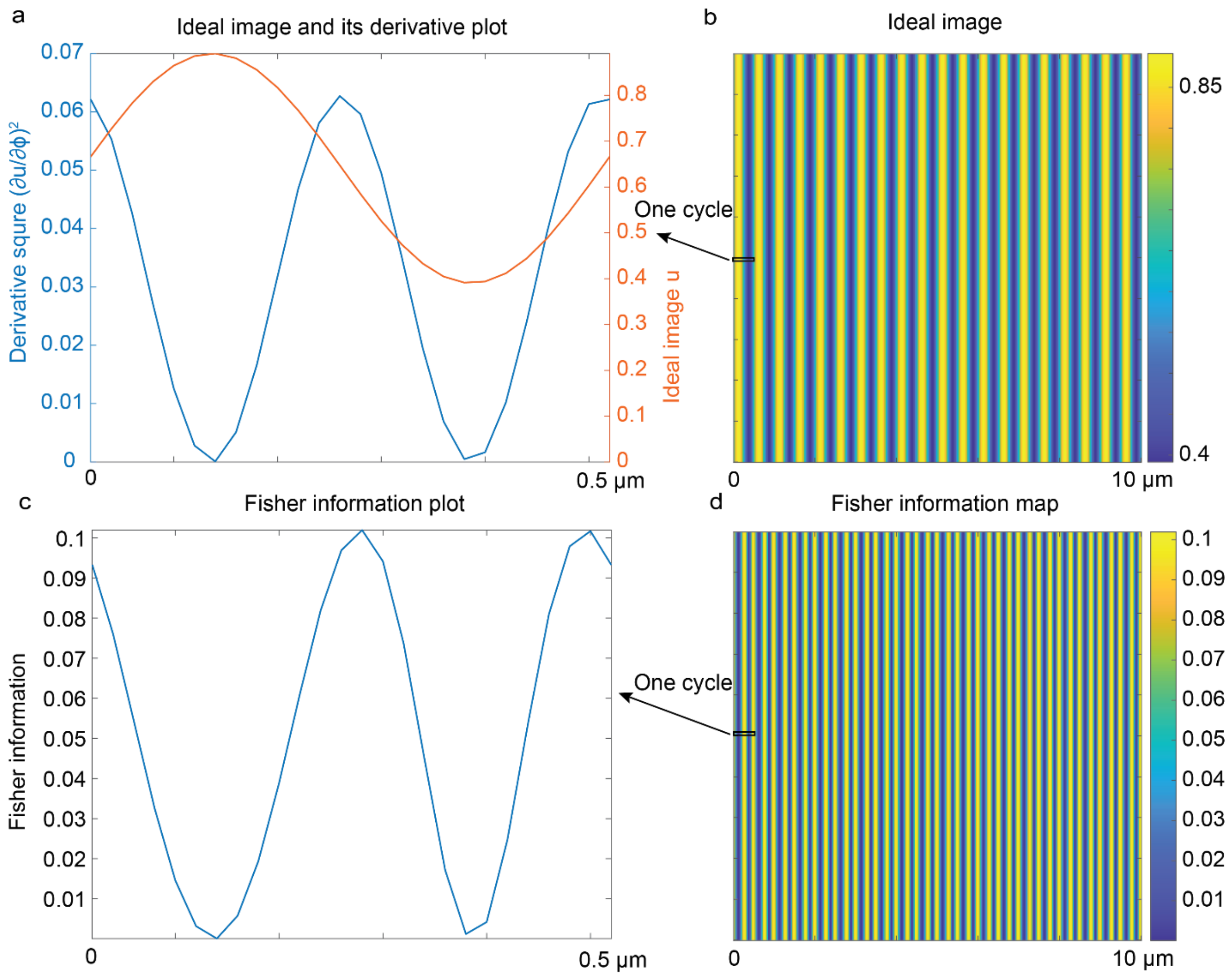

Supplementary Fig. 1. Fisher information $I(\phi)$ calculation visualization. (a) Ideal image and its derivative plotted in one cycle. (b) Ideal image of a sinusoidal grating object in wide field microscope. (c) Fisher information (unit: rad^{-2}) distribution in one cycle of the sinusoidal pattern. (d) Fisher information map from a wide-field microscope. The sinusoidal grating object had 500*500 pixels with pixel size 0.02 μm, a frequency of $k = \frac{NA}{\lambda}$. Signal photon emission density was 5000 photons/μm^2. Fisher information $I(\phi) = \sum_x \frac{1}{u(x)} \left(\frac{\partial u(x)}{\partial \phi}\right)^2$ is periodic with interval $\frac{1}{k}$.

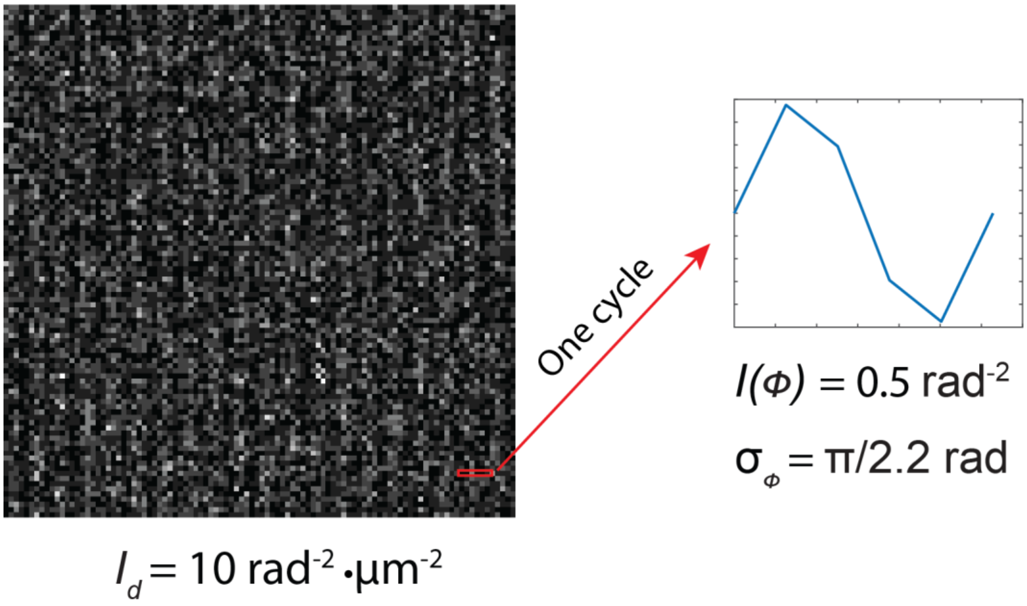

Supplementary Fig. 2. Grating object at frequency $k = \frac{NA}{\lambda}$ and plot of expected pixel value of one cycle. The length of one cycle of the sine pattern is 0.5 µm, and the pixel size is 0.1 µm. Applying threshold $I_d = 10\, rad^{-2} \cdot \mu m^{-2}$ to a unit area (one cycle of the sinusoidal wave), the Fisher information corresponds to phase estimation uncertainty $\sigma_\phi = \frac{\pi}{2.2}$. This threshold was then used as a predetermined critical point distinguishing resolved and unresolved cases in this work.

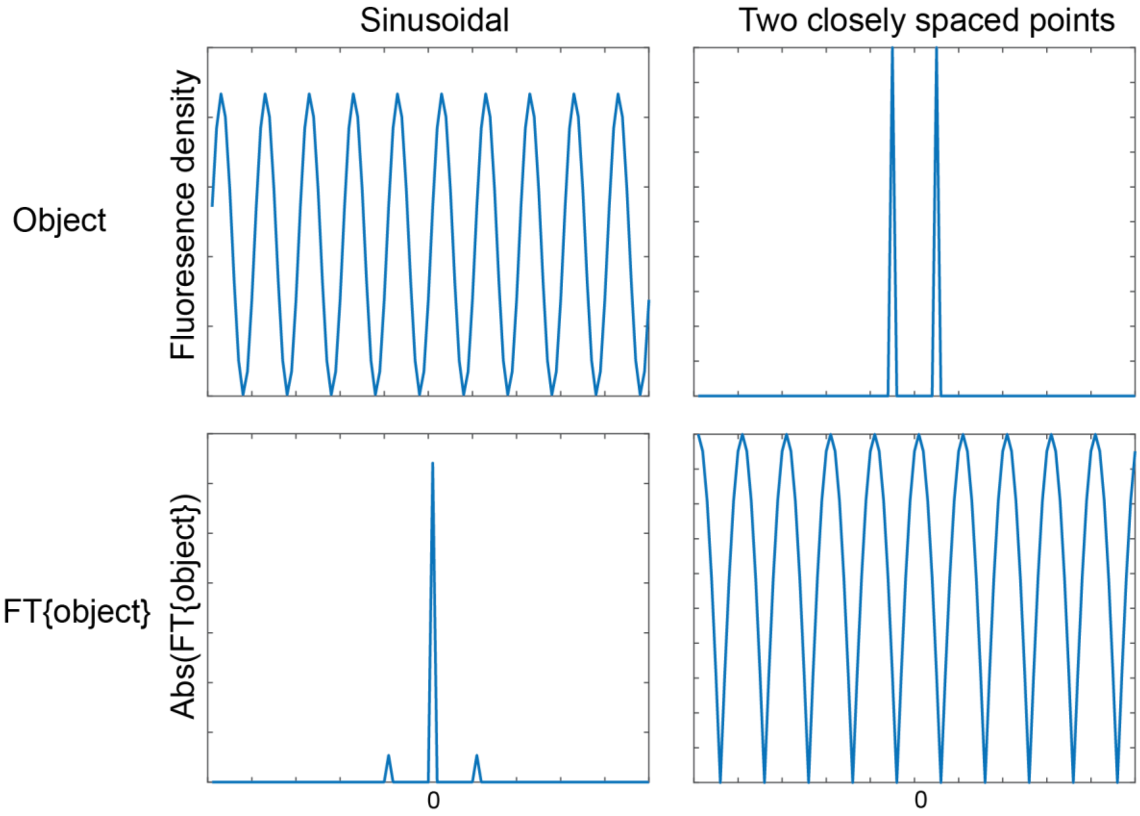

Supplementary Fig. 3. Sinusoidal pattern and two close point emitters and their corresponding magnitude of Fourier transform in 1D. The Fourier transform of sinusoidal pattern has one pair of non-DC components. The magnitude of the Fourier transforms of two point emitters spread across the entire spatial frequency range.

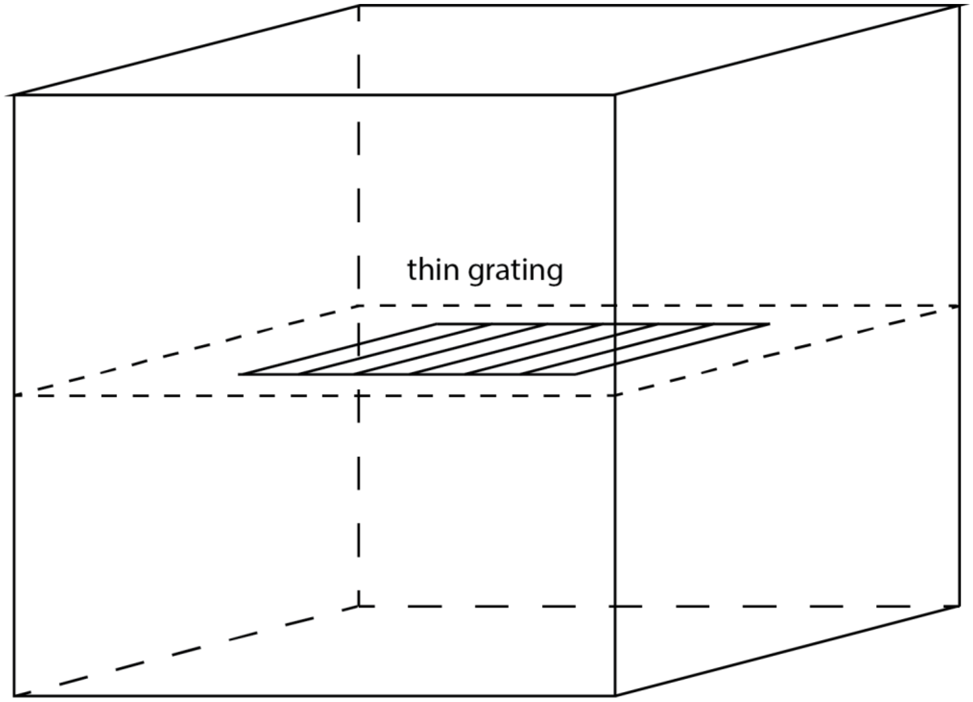

Supplementary Fig. 4. Volumetric specimen simulation model. A thin sine-pattern grating is immersed in a 3D volume with uniformly distributed background fluorescence. The thin grating was assumed to be zero thickness and in the focus plane of the imaging system.

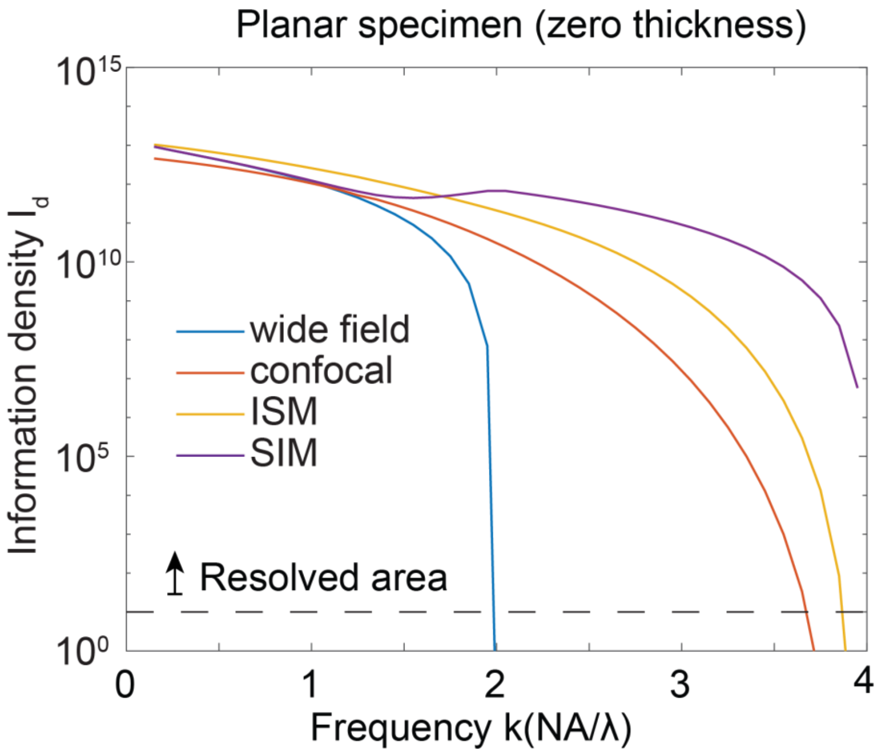

Supplementary Fig. 5. Information density I_d demonstrated for four imaging modalities in thin sample imaging at infinite photons (approach with 50 trillion photons/μm^2). The simulation conditions were set with numerical aperture of 1.4, immersion medium refractive index of 1.5, and emission wavelength of 0.7 μm. Confocal system considered a pinhole diameter of 0.5 AU. ISM considered a detector pixel size 0.26 AU with 5 by 5 pixels, covering detector area 1.3 AU × 1.3 AU. SIM implemented a structured illumination frequency of $k_{st} = \frac{2NA}{\lambda}$, with nine illumination patterns in 3 illumination orientations, one aligning with the sine pattern object. Each illumination orientation has three phase patterns. Camera pixel size in the wide-field system and SIM equals scanning intervals in the confocal system and ISM and they were set to 0.1 μm (0.16 AU). Photon collection efficiencies were considered based on 4Pi solid angle emission, objective NA and the pinhole rejection. The above simulation had photon collecting efficiency of 32.05% for wide-field and SIM microscopy, 10.77% for confocal microscopy with a 0.5 AU pinhole, and 24.68% for ISM with a 1.3 AU FOV.

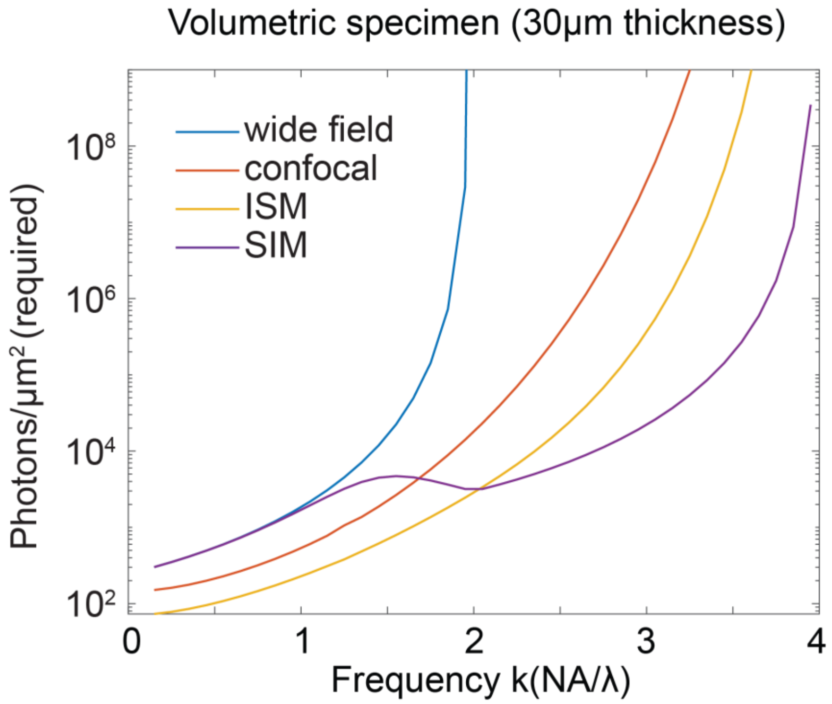

Supplementary Fig. 6. Photons per area required for different grating structures to reach the applied resolving criterion ($I_d = 10\ rad^{-2} \cdot \mu m^{-2}$) in volumetric specimen, ranging from zero to one hundred million photons per μm^2. The simulation conditions are set with signal photon emission density of 5000 photons/μm^2, background photon emission density 500 photons/μm^3, numerical aperture of 1.4, immersion medium refractive index of 1.5, and emission wavelength of 0.7 μm. Confocal system considered a pinhole diameter of 0.5 AU. ISM considered a detector pixel size 0.26 AU with 5 by 5 pixels, covering detector area 1.3 AU × 1.3 AU. SIM implemented a structured illumination frequency of $k_{st} = \frac{2NA}{\lambda}$. SIM uses 9 illumination patterns with 3 illumination orientations each having 3 phase patterns. One of SIM's illumination patterns aligned with the sine pattern object. Camera pixel size in the wide-field system and SIM equal scanning intervals in the confocal system and ISM and they were set to 0.1 μm (0.16 AU). Photon collection efficiencies were considered based on 4Pi solid angle emission, objective NA and the pinhole rejection. The above simulation had photon collecting efficiency 32.05% for wide-field and SIM microscopy, 10.77% for confocal microscopy with a 0.5 AU pinhole, and 24.68% for ISM with a 1.3 AU FOV.

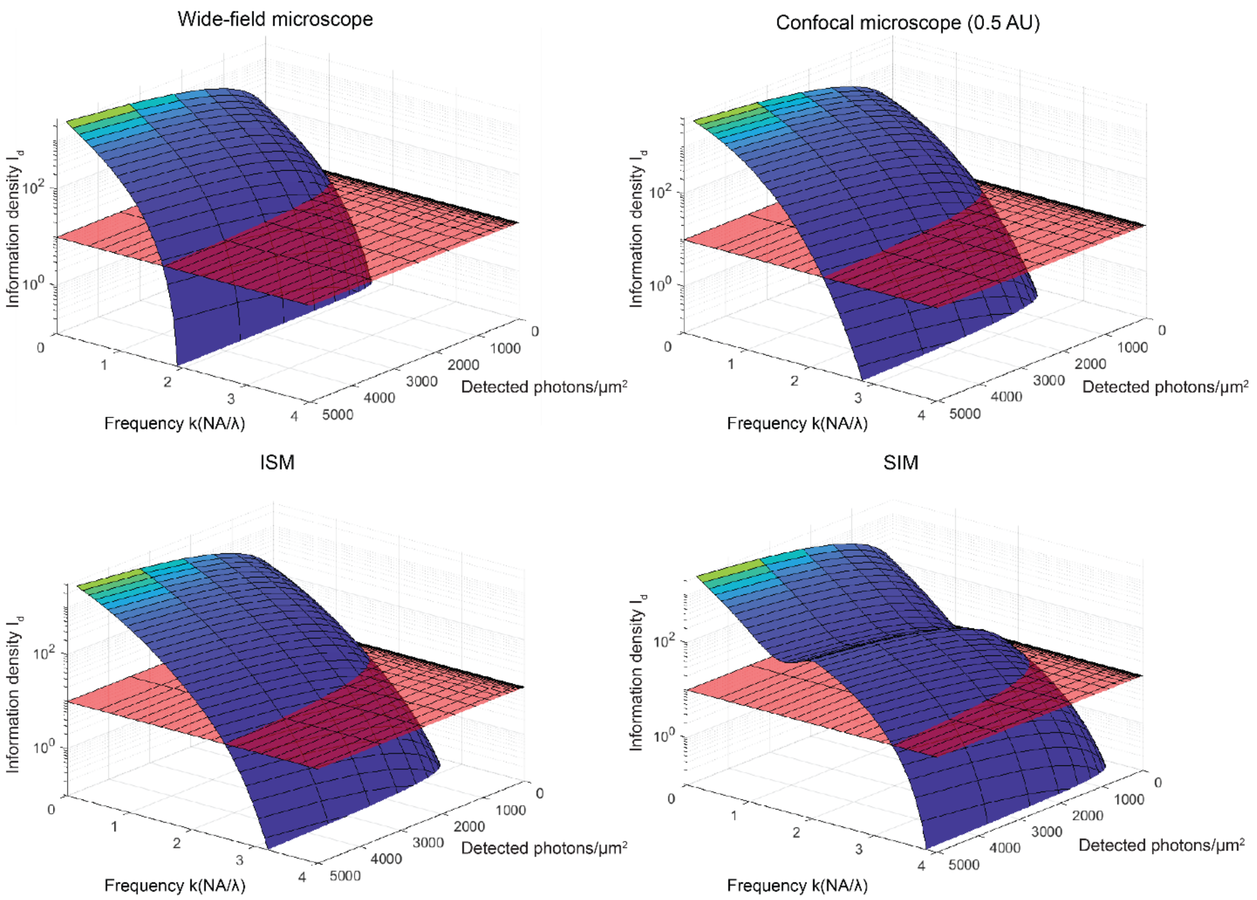

Supplementary Fig. 7. Fisher information calculation in four imaging modalities with respect to detected photons and frequency. Red planes indicated the resolving criterion $I_d = 10\, rad^{-2} \cdot \mu m^{-2}$. The simulation conditions were conducted in planar specimen (background free), numerical aperture of 1.4, immersion medium refractive index of 1.5, and emission wavelength of 0.7 μm. The confocal system is configured with a pinhole diameter of 0.5 AU. ISM modality is set with a detector pixel size of 0.26 AU with 5 by 5 pixels, covering a 1.3 AU square. SIM employs a structured illumination frequency of $k_{st} = \frac{2NA}{\lambda}$, with nine illumination patterns in 3 illumination orientations, one aligning with the sinusoidal pattern object. Each illumination orientation had three phase patterns. The camera pixel size for wide-field system and SIM, as well as the scanning intervals for confocal and ISM, were set to 0.1 μm (0.16 AU).

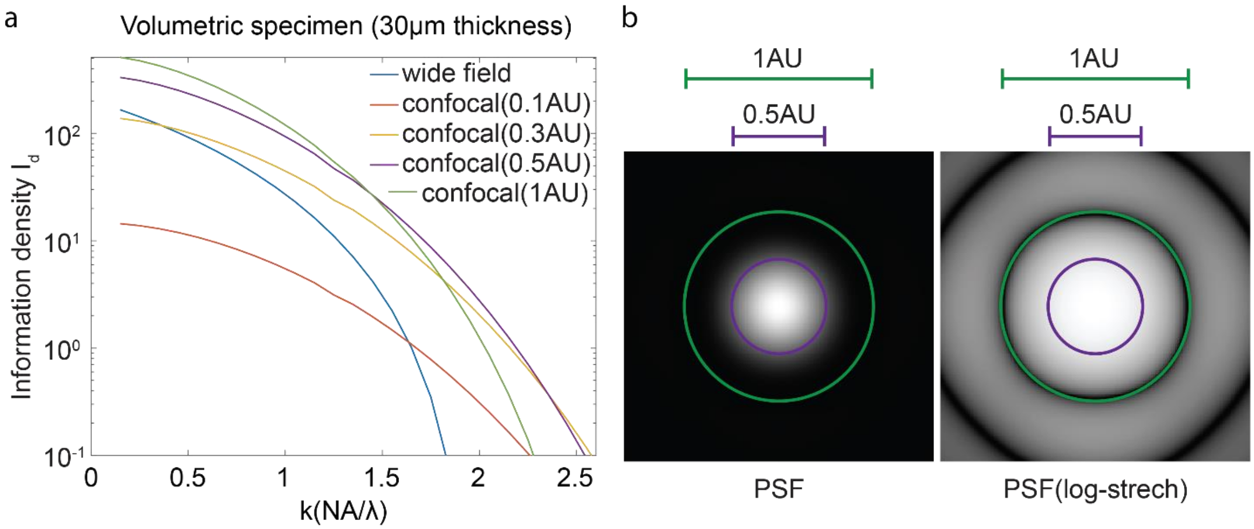

Supplementary Fig. 8. Information density I_d in a confocal system with different circular pinhole diameters. (a) Comparison of information density I_d between a wide-field system and confocal systems with different pinhole diameters. (b) Visualization of size comparison between excitation PSF as mapped on the image plane and pinhole. The simulation conditions are in volumetric specimen with thickness of 30 μm set with signal photon emission density of 5000 photons/μm^2, background photon emission density 500 photons/μm^3, numerical aperture of 1.4, immersion medium refractive index of 1.5, and emission wavelength of 0.7 μm. Photon collection efficiencies are considered based on 4Pi solid angle emission, objective NA and the pinhole rejection. The above simulation has photon collecting efficiency of 32.05% for wide-field, 0.66%, 4.61%, 10.77%, 21.12%, 26.79% for confocal microscopy with a 0.1 AU, 0.3 AU, 0.5 AU, 1 AU pinhole respectively.

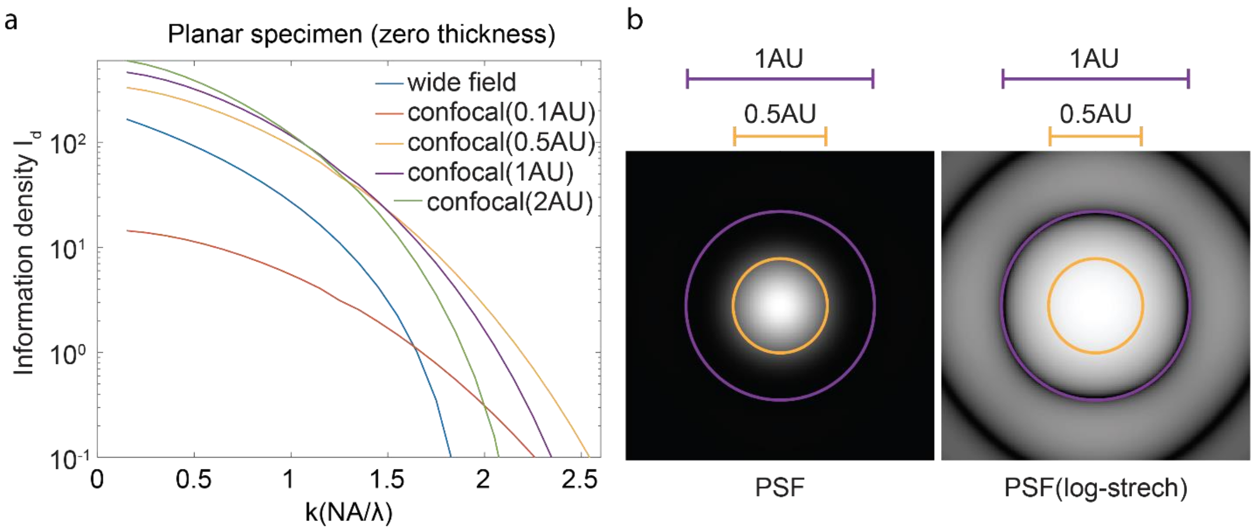

Supplementary Fig. 9. Information density $\boldsymbol{I_d}$ in a confocal system with different circular pinhole diameters. (a) Comparison of information density $\boldsymbol{I_d}$ between a wide-field system and confocal systems with different pinhole diameters. (b) Visualization of size comparison between excitation PSF as mapped on the image plane and pinhole. The simulation conditions are in planar specimen set with signal photon emission density of 5000 photons/μm^2, numerical aperture of 1.4, immersion medium refractive index of 1.5, and emission wavelength of 0.7 μm. Photon collection efficiencies are considered based on 4Pi solid angle emission, objective NA and the pinhole rejection. The above simulation has photon collecting efficiency of 32.05% for wide-field, 0.66%, 10.77%, 21.12%, 26.79% for confocal microscopy with a 0.1 AU, 0.5 AU, 1 AU, 2 AU pinhole respectively.

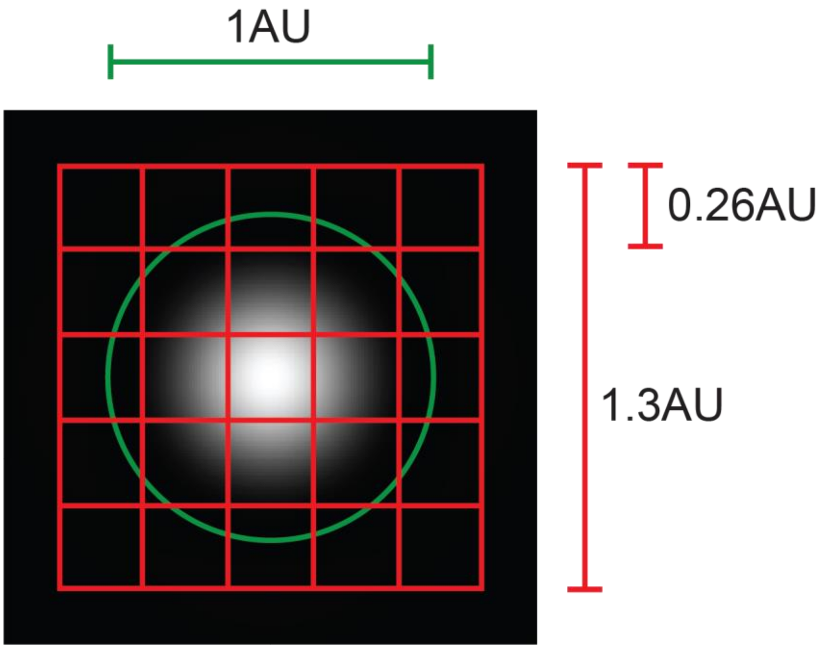

Supplementary Fig. 10. Size comparison between excitation PSF and ISM detector pixel and FOV. ISM is set with a detector pixel size of 0.26 AU with 5 by 5 pixels, covering a 1.3 AU square, which is in accordance with common usage (*44*).

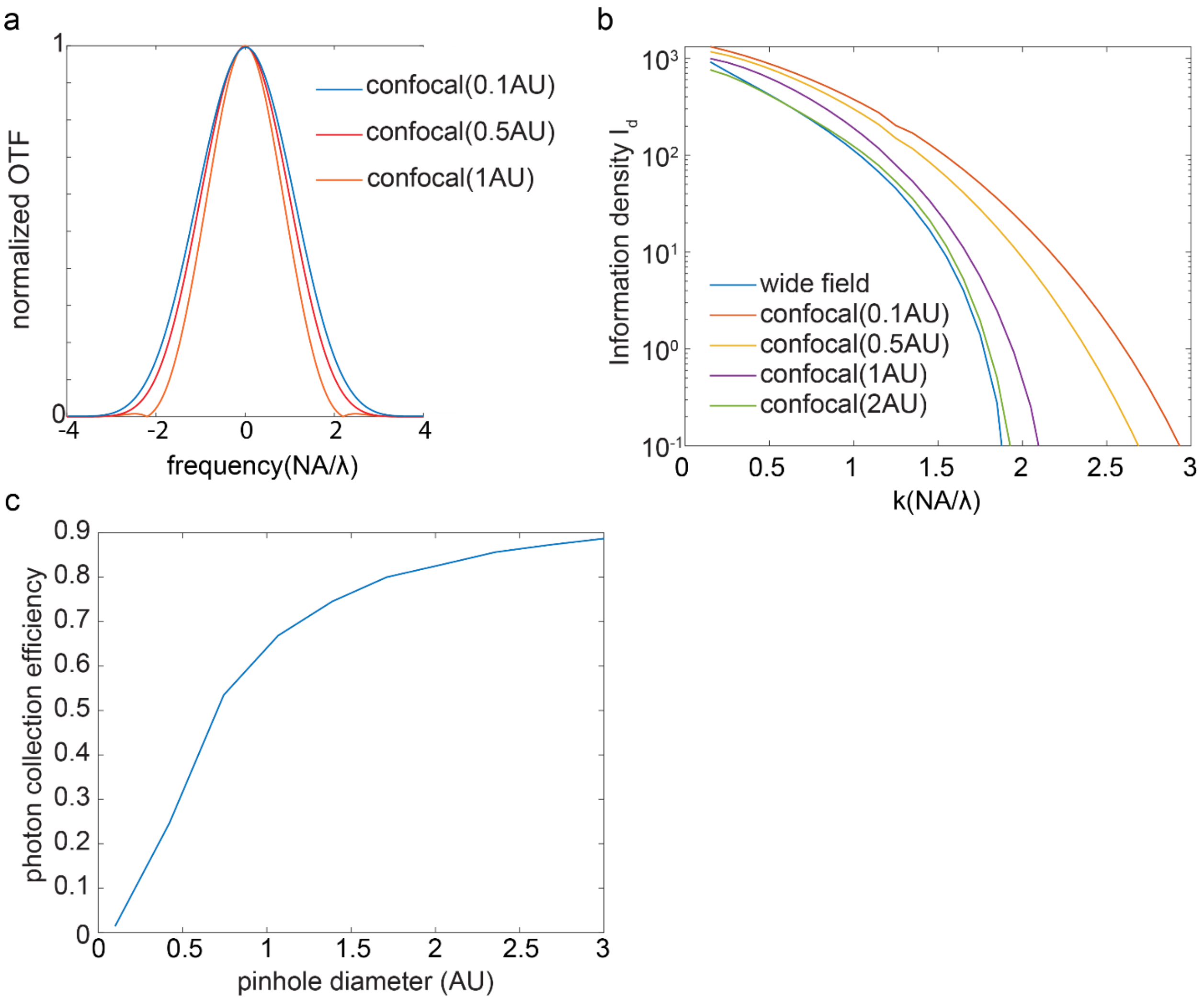

Supplementary Fig. 11. Information density of confocal systems, optical transfer function, and photon collection efficiency with respect to its pinhole diameters**.** (a) Normalized effective OTF of confocal microscope with different pinhole diameters. (b) Information density $\boldsymbol{I_d}$ with a *fixed detected* photon count from confocal systems with different pinhole diameters. Smaller pinhole size provides better resolving power if receiving the same number of photons due to extension in OTF. (c) Photon collection efficiency with respect to pinhole diameter. Plots in (b) and (c), are simulated under the condition: FOV 10 μm × 10 μm (16.4 AU × 16.4 AU), NA=1.4, emission wavelength λ=0.7 μm, immersion media refractive index n=1.5, and pixel size in wide-field system as well as scanning interval in confocal system 0.1 μm (0.16 AU).

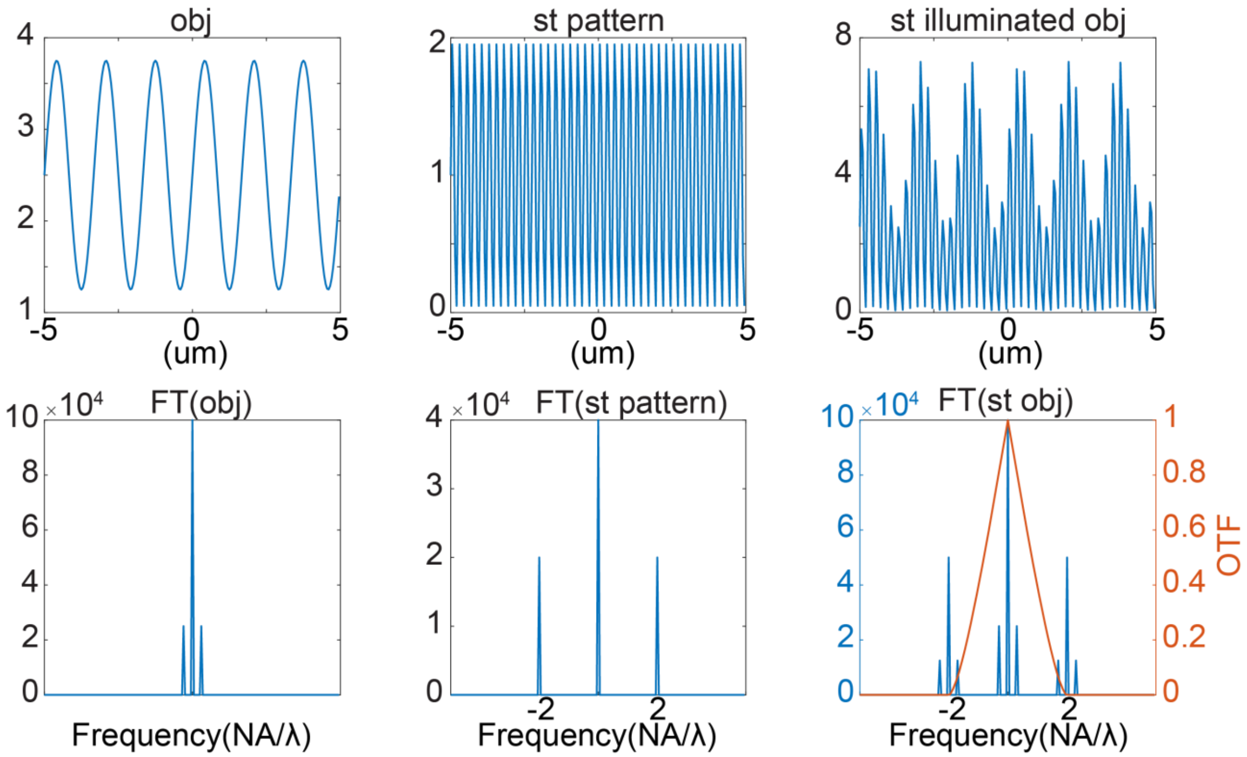

Supplementary Fig. 12. Structured illumination microscope image visualized in 1d when illuminated with sin pattern when object is of low frequency. From left to right, panels show fluorophores distribution, structured illumination pattern intensity distribution and fluorescence distribution of the structurally illuminated object. Top panels are in real space while bottom panels are magnitude in Fourier space.

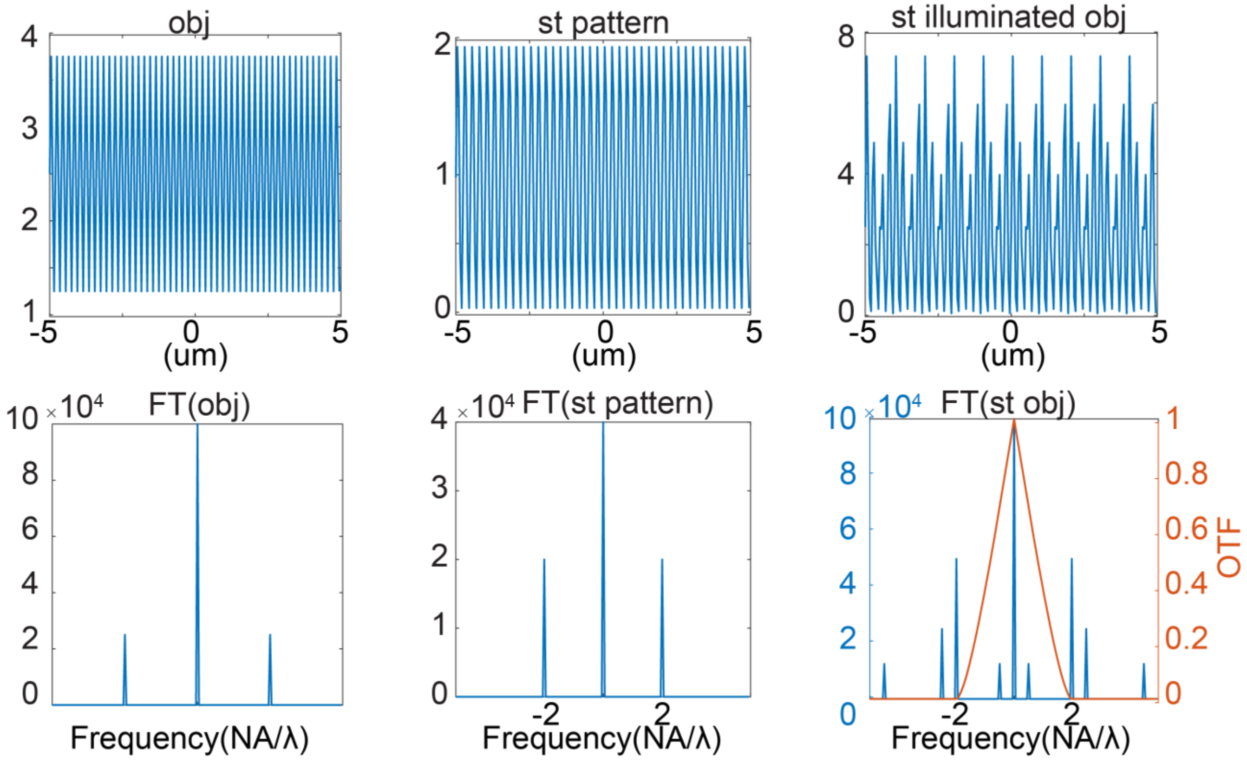

Supplementary Fig. 13. Structured illumination microscope image visualized in 1d when illuminated with sin pattern when object is of high frequency. From left to right, panels show fluorophores distribution, structured illumination pattern intensity distribution and fluorescence distribution of the structurally illuminated object. Top panels are in real space while bottom panels are magnitude in Fourier space.

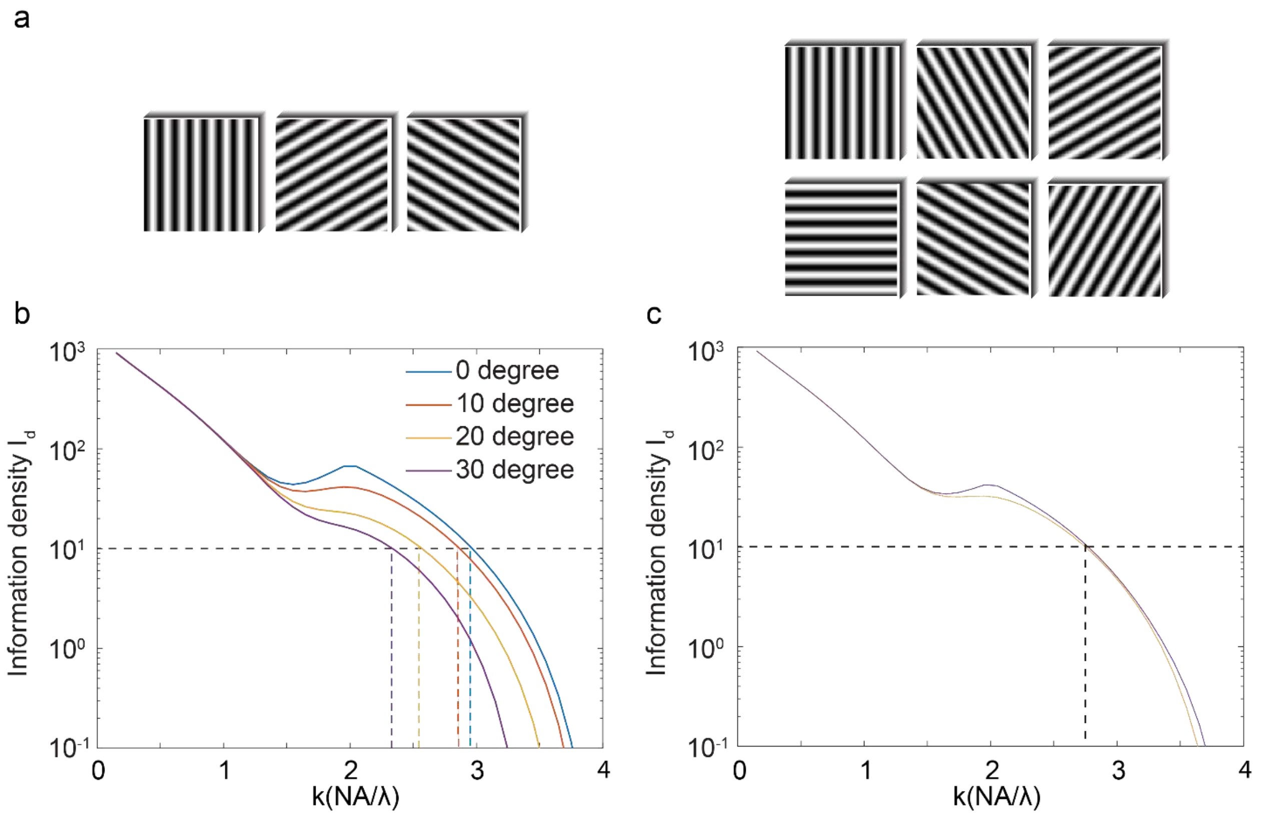

Supplementary Fig. 14. SIM information density I_d with respect to its initial orientation of the structured illumination patterns set. (a) Structured illumination patterns. Each icon (structured illumination pattern of one orientation) contains patterns of 3 phases. (b) Information density I_d of SIM with structured illumination patterns containing 3 orientations. The information density of SIM using four different sets of illumination patterns (angle indicates the degree of a clockwise rotation for the initial illumination angles) are shown. (c) Information density I_d of SIM with structured illumination patterns containing 6 orientations. Plots in (b) and (c) are simulated under the condition: FOV: 10 μm × 10 μm (16.4 AU × 16.4 AU), NA=1.4, emission wavelength λ=0.7 μm, immersion media refractive index n=1.5, and camera pixel size 0.1 μm (0.16 AU). Photon density is 5000 photons/μm^2. Photon collection efficiencies were considered based on 4Pi solid angle emission, objective NA and the pinhole rejection. The above simulation had photon collecting efficiency of 32.05% for SIM.

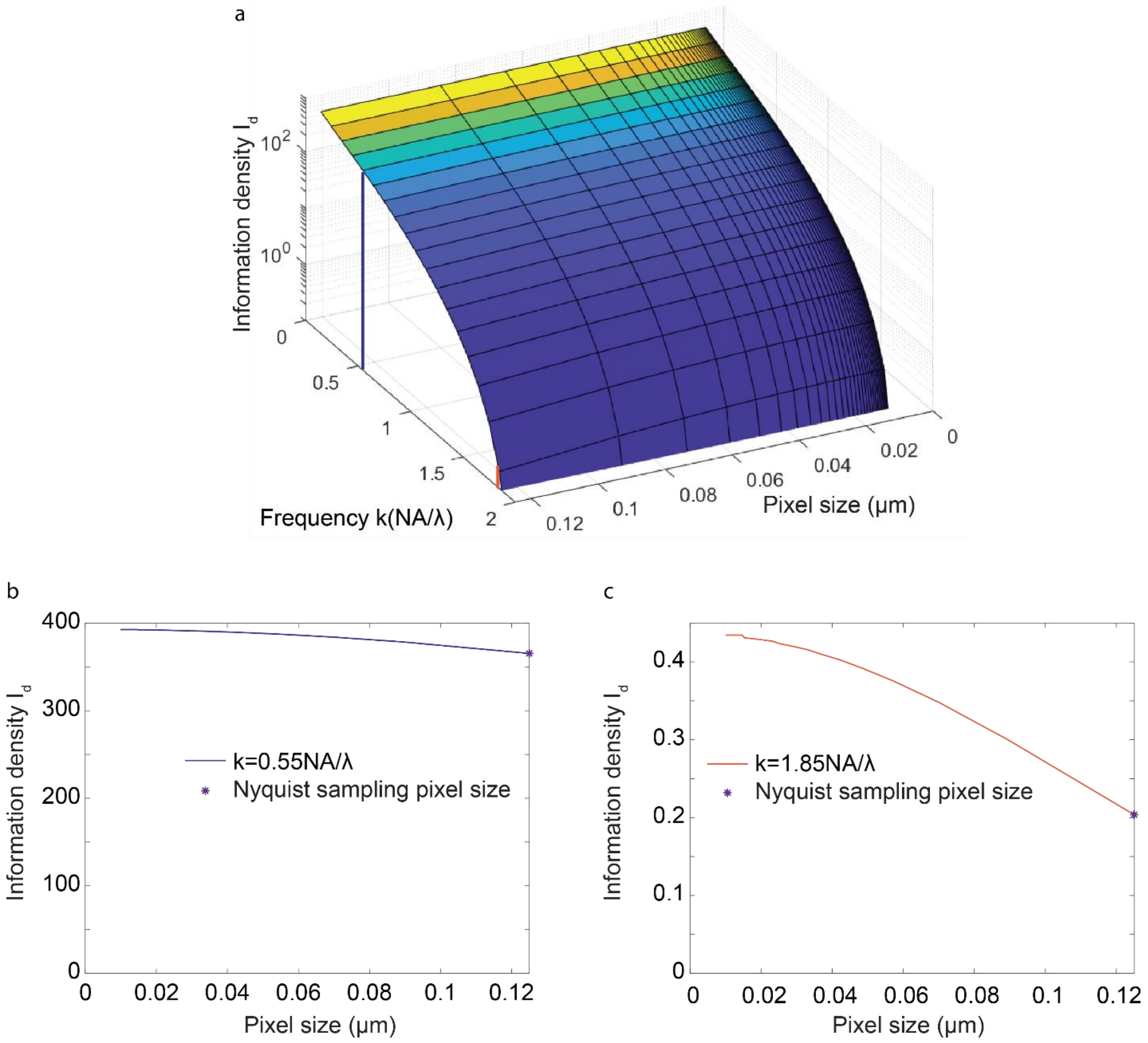

Supplementary Fig. 15. Influence camera pixel size on information density on wide-field microscope. (a) Surface plot of information density versus sinusoidal grating's frequency and pixel size up to Nyquist requirement ($\frac{\lambda}{4NA} = 0.125\mu m$). (b) Plots of information density versus pixel size at low frequency. (c) Plots of information density versus pixel size at low frequency. In the above plots, the photon emission density was set as 5,000 photons/μm^2, with a numerical aperture of 1.4, an immersion medium refractive index of 1.5, and an emission wavelength of 0.7 μm. Photon collection efficiencies were considered based on 4Pi solid angle emission, objective NA and the pinhole rejection. The above simulation had photon collecting efficiency 32.05% for wide-field.
